# Comprehensive Experimental and Computational Characterization of a Phenylacetamide‐Based Molecule

**DOI:** 10.1155/bmri/2415605

**Published:** 2026-07-14

**Authors:** Tugba Agbektas, Farid N. Naghiyev, Burak Tüzün, Ali N. Khalilov, Ayca Tas, Cemile Zontul, Unal Ozum, Yavuz Silig, Alireza Poustforoosh, Ibrahim G. Mamedov

**Affiliations:** ^1^ Department of Food Processing, Food Technology Program, Yıldızeli Vocational School, Sivas Cumhuriyet University, Sivas, Turkey, cumhuriyet.edu.tr; ^2^ Department of Chemistry, Baku State University, Baku, Azerbaijan, bsu.edu.az; ^3^ Plant and Animal Production Department, Technical Sciences Vocational School of Sivas, Sivas Cumhuriyet University, Sivas, Turkey, cumhuriyet.edu.tr; ^4^ Composite Materials Scientific Research Center, Azerbaijan State Economic University (UNEC), Baku, Azerbaijan; ^5^ Azerbaijan State Oil and Industry University, Baku, Azerbaijan, asoiu.edu.az; ^6^ Department of Chemistry, Washington University, Saint Louis, Missouri, USA, wustl.edu; ^7^ Department of Biochemistry, Faculty of Medicine, Sivas Cumhuriyet University, Sivas, Turkey, cumhuriyet.edu.tr; ^8^ Department of Chemistry and Chemical Processing Technologies Services, Yıldızeli Vocational School, Sivas, Turkey; ^9^ Department of Neurosurgery, Faculty of Medicine, Sivas Cumhuriyet University, Sivas, Turkey, cumhuriyet.edu.tr; ^10^ Medicinal and Natural Products Chemistry Research Center, Shiraz University of Medical Sciences, Shiraz, Iran, sums.ac.ir

**Keywords:** ADME/T, biochemical analyses, gene expression, molecular docking, neuroblastoma cancer

## Abstract

The aim of this study was to synthesize Tetrahydroisoquinoline Derivative 1 (M1) and to evaluate its biological activities in the SH‐SY5Y neuroblastoma cell line. Theoretical calculations for the investigated molecule were performed using the Gaussian software package at the B3LYP, HF, and M062X levels with the 6‐31g, 6‐31++g, and 6‐31++g(d,p) basis sets. Subsequently, the activity of the compound against SH‐SY5Y cancer–related proteins (PDB IDs: 2F37, 3PBL, and 5WIV) was assessed. In addition, the molecule‐likeness properties of the molecule were evaluated through ADME/T analyses. The cytotoxic activity of M1 in the SH‐SY5Y cell lines was determined using the MTT assay. Following treatment with M1, the expression levels of apoptosis‐related genes (MYC, CASP2, BAX, and NF‐*κ*B1) and genes associated with DNA repair mechanisms (TP53, RAD51, BRCA2, and MDM2) were analyzed by RT‐PCR. Enzyme activities were also measured in M1‐treated SH‐SY5Y cells. The results demonstrated that M1 exerted its highest cytotoxic effect in the SH‐SY5Y cell line after 72 h of incubation. Compared with the control group, M1 showed a stronger effect on G6PDH activity in SH‐SY5Y cells, while catalase activity increased by 78% following M1 treatment. Moreover, M1 markedly reduced cell viability in SH‐SY5Y cells relative to the control group. In conclusion, these findings indicate that Tetrahydroisoquinoline Derivative M1 exhibits pronounced cytotoxic activity in SH‐SY5Y neuroblastoma cells and significantly modulates oxidative stress–related enzyme activities as well as the expression of genes involved in apoptosis and DNA repair pathways, suggesting that M1 may represent a novel and promising candidate for neuroblastoma therapy.

## 1. Introduction

**Scheme 1:**
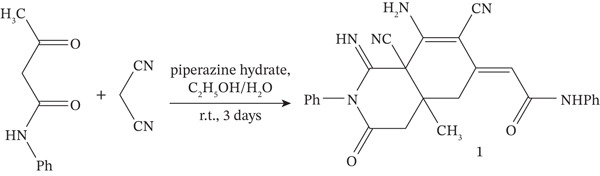
The synthesis of M1.

Cancer and neurodegenerative diseases represent a growing public health challenge due to their rising global incidence. Due to their structural adaptability and ability to modulate several cancer‐related pathways, isoquinoline derivatives represent a key class of multitarget therapeutic agents. Several natural and synthetic isoquinoline alkaloids (ellipticine, palmatine, berberine, etc., analogs) exhibit significant anticancer effects on neuroblastoma (NB) cells, as extensively reviewed in the literature [1–4]. Inspired by these reports, our work employs acetoacetanilide in multicomponent reactions to access a variety of heterocyclic systems, notably isoquinoline derivatives [2–5]. Here, we present the biological evaluation of the compound, complementing its synthesis (Scheme 1) and crystallographic characterization reported previously [6].

NB is a type of neuroblastic tumor that arises from primitive neural crest cells located in the sympathetic nervous system, with 65% of cases originating from the adrenal medulla [7, 8]. While NB constitutes 8%–10% of childhood cancers, it represents approximately 15% of cancer‐related fatalities among children [9]. NB symptoms begin to appear in children aged 18 months, and 90% of them are diagnosed after the age of 10 [10]. Approximately 40% of patients diagnosed with NB are classified in the high‐risk group [11]. Various treatment methods, such as surgery and induction chemotherapy, autologous peripheral blood stem cell support, radiation, biological agents, and immunotherapy, are applied to high‐risk NB patients [12, 13]. Despite intensive treatment, children diagnosed with high‐risk NB are resistant to treatment, and their overall survival rate is approximately 50% [8]. Enhanced induction chemotherapy correlates with better overall survival rates [14, 15]. Doxorubicin, cyclophosphamide, cisplatin, and etoposide are used as chemotherapeutic agents [16–20]. Despite current therapeutic advances and research, there is an unpredictable clinical situation for advanced NB. Rapid development of new strategies and targeted therapies is needed to cure the disease [21]. Apoptosis refers to a form of programmed cell death that involves genetic pathways crucial for development and the maintenance of homeostasis in healthy tissues. Apoptosis is important for maintaining tissue homeostasis in a multicellular organism [22]. Even in cases where very few cells undergo apoptosis, tumor formation may occur even if there is no proliferation rate [23]. Delayed activation of apoptotic pathways is an important phenomenon for NB regression and therapy resistance. Avoidance of apoptosis is not only involved in the gradual process of tumorigenesis but is also one of the hallmarks of human cancers, including NB. Current treatment regimens of cancer cells, namely, the response to chemo‐, radio‐, or immunotherapy, are largely due to the induction of apoptosis in neoplastic cells, combining resistance to apoptosis and resistance to therapy for response to cytotoxic treatments [24–26]. Dysregulation of cell cycle progression is seen in many human cancers. Accordingly, cellular processes such as proliferation, differentiation, and apoptosis may be affected, resulting in tumorigenesis [27]. Antitumor growth drugs that “accelerate” or “brake” excessive cell turnover may be a powerful treatment option for unfavorable NBs [28].

Upon a thorough examination of the most recent studies, it becomes evident that theoretical calculations and experimental methodologies produce consistent results that mutually reinforce one another [29]. This scenario started to be utilized for the synthesis and design of more active and effective molecules in theoretical research conducted prior to experimental investigations [30]. Docking study is one of the most frequently employed techniques for comparing the biological activities of various molecules [31]. The molecular docking technique is employed to evaluate the efficacy of various molecules in relation to enzymes, aiming to identify those with the greatest biological activity. Experimental studies are guided by these calculations. For the theoretical calculations of the studied molecule, calculations were made at B3LYP, HF, and M062X [32–34] levels on the 6‐31g, 6‐31++g, and 6‐31++g(d,p) basis sets using the Gaussian package program. Afterwards, the activity of the studied molecule against the SH‐SY5Y cancer protein (PDB ID: 2F37, 3PBL, and 5WIV) [35–37] was examined. Ultimately, the characteristics of the M1 molecule associated with the molecule under investigation were assessed through the execution of ADME/T analysis.

This study is among the limited number of investigations that address the biological effects of an isoquinoline‐derived compound on NB cells within an integrated framework combining experimental biochemical analyses and molecular docking approaches. Although numerous studies have reported the anticancer potential of isoquinoline alkaloids, the majority of these investigations are either restricted to experimental cytotoxicity data or fail to establish a direct link between theoretical calculations and biological validation. One of the most significant aspects of the present study is that the isoquinoline derivative, whose synthesis and crystallographic characterization were previously reported, was evaluated for the first time in a NB (SH‐SY5Y) cell line by analyzing expression changes in key apoptosis‐related genes (*MYC*, *CASP2*, *BAX*, and *NF-κB1*), as well as cell cycle–associated genes of major relevance in cancer biology (*TP53*, *RAD51*, *BRCA2*, and *MDM2*). In addition, the effects of the isoquinoline derivative on antioxidant defense mechanisms in SH‐SY5Y cells were investigated by measuring the activities of glutathione (GSH), glutathione S‐transferase (GST), glucose‐6‐phosphate dehydrogenase (G6PDH), catalase (CAT), and superoxide dismutase (SOD).

## 2. Materials and Methods

### 2.1. Chemistry Section

Melting points were measured using a Stuart SMP30 melting point apparatus, and no corrections were applied. The 1H and 13C NMR spectra of established chemicals were obtained using a Bruker Avance II+300 (UltraShieldTM Magnet) instrument, with measurements conducted in DMSO at frequencies of 300 and 75 MHz, respectively. With solvent resonance in DMSO‐d6 solutions serving as the internal standard, chemical shifts from tetramethylsilane were reported in parts per million. All chemicals available for commercial purchase were obtained from Merck (Darmstadt, Germany) and Fluka (Buchs, Switzerland) and utilized without additional purification. The reactions were observed using TLC on silica gel 60 f254 plates.

The target compound (1) was synthesized via a multicomponent reaction involving acetoacetanilide, malononitrile, and piperazine hydrate in an ethanol–water medium. The reaction proceeds through condensation and subsequent cyclization steps at room temperature, affording the isoquinoline‐based phenylacetamide derivative. Detailed experimental conditions are provided in the supporting information file and Figures S1, S2, S3 and S4.

### 2.2. Cell Culture

SH‐SY5Y NB cells were maintained at 37°C in an incubator with 5% CO_2_ in DMEM supplemented with 10% fetal bovine serum (FBS) and 100 U/mL penicillin. Cells were routinely passaged, and the culture medium was refreshed upon reaching the appropriate confluence.

### 2.3. In Vitro Cytotoxicity Determination (MTT [(3‐(4,5‐Dimethylthiazol‐2‐yl)‐2,5‐Diphenyl Tetrazolium Bromide)])

Cells were seeded into 96‐well plates at 1 × 10^4^ cells/well, a density selected based on preliminary optimization to ensure appropriate confluence and a linear MTT response over 24–72 h, consistent with commonly used SH‐SY5Y MTT protocols. After 24 h, cells were treated with M1 at concentrations ranging from 1 to 100 *μ*M and incubated for the indicated exposure times. Cell viability was assessed using the MTT assay [38, 39]. Following incubation with MTT, the supernatant was aspirated, and 100 *μ*L DMSO was added to each well to dissolve the formazan crystals. Plates were incubated for 15 min, and absorbance was measured at 570 nm. IC_50_ values were calculated using GraphPad Prism 7. All experiments were performed in triplicate (*n* = 3), and results are presented as the mean of three independent measurements.

### 2.4. Extraction of RNA From Cell Culture Samples

The SH‐SY5Y cell line was retreated with the IC_50_ dose of the M1 molecule, determined by the MTT assay at 48 h. Following an additional 48‐h incubation period, RNA isolation was performed in accordance with the RNeasy Plus Mini Kit (Qiagen, Germany, Cat No.: 74104) protocol. RNA isolation was performed from cells given the M1 molecule, from cells given the M1 molecule solution, and from control cells that received nothing.

### 2.5. Complementary DNA (cDNA) Synthesis

To determine the expression levels of apoptosis‐related genes (*MYC*, *CASP2*, *BAX*, and *NF-κB1*) and genes associated with cell regulation/DNA repair mechanisms (*TP53*, *RAD51*, *BRCA2*, and *MDM2*), cDNA was synthesized from RNA in accordance with the manufacturer′s kit (RT First Strand Kit [Qiagen, Germany, Cat. No.: 330404]) protocol for subsequent RT‐PCR analysis.

### 2.6. Real‐Time PCR Analysis

Utilizing the enhanced RT^2^ SYBR Green qPCR Master Mix Kit, the expression of apoptosis (*MYC*, *CASP2*, *BAX*, and *NF-κB1*) and cell cycle (*TP53*, *RAD51*, *BRCA2*, and *MDM2*) genes (RT^2^ qPCR Primer Assay [Qiagen, Germany]) was evaluated using the *Δ* *Δ*_CT_ technique on an RT‐PCR equipment. *GAPDH* was used for housekeeping. RT‐qPCR experiments were performed in triplicate (*n* = 3) for each experimental group, and the reported values represent the mean of three independent measurements. In this investigation, SYBR Green was utilized as a fluorescent dye. A 25 *μ*L qPCR mix was made from samples containing cDNA in accordance with the kit instructions, and statistical calculation of the results acquired in the device was accomplished by use of the *Δ* *Δ*_CT_ method utilizing https://dataanalysis2.qiagen.com/pcr software.

### 2.7. Determination of Antioxidant Levels

#### 2.7.1. Glutathione (GSH) Determination

SH‐SY5Y cells were plated in six‐well plates and subsequently treated with the M1 molecule. The cells were trypsinized, and the supernatant was removed after 48 h. A volume of 250 *μ*L was obtained from each cell sample, and the procedure was followed. The methodology that was suggested by Giustarini et al. [40] was used to determine the GSH level. The protocol proposed by Giustarini et al. is designed for the determination of GSH and glutathione disulfide (GSSG) concentrations in blood, cell lysates, and various tissues. The concentration of GSSG is subsequently quantified spectrophotometrically using the GSH recycling assay, which is based on the reduction of GSSG to GSH by GSH reductase in the presence of NADPH, followed by its reaction with 5,5 ^′^‐dithiobis‐(2‐nitrobenzoic acid).

#### 2.7.2. Glutathione‐S‐Transferase (GST) Determination

One hundred and fifty microliters was taken from each of the M1 samples treated with SH‐SY5Y cell lines, and the methodology suggested by Ghelfi et al. [41] was used to determine the GST level. According to this method, the assay is based on the conjugation of reduced GSH with the substrate 1‐chloro‐2,4‐dinitrobenzene (CDNB). The reaction mixture consisted of potassium phosphate buffer (0.1 M, pH 6.5), GSH, CDNB, and an appropriate amount of sample. Formation of the GS–DNB conjugate was monitored by measuring the increase in absorbance at 340 nm at 25°C. Enzyme activity was calculated using the molar extinction coefficient of the conjugate and expressed as nanomoles per minute per milligram of protein. Protein concentrations were determined prior to the assay, and all measurements were performed under conditions in which the reaction rate was linear with respect to time and protein concentration [41].

#### 2.7.3. Determination of Glucose‐6‐Phosphate Dehydrogenase (G6PDH) Enzyme Activity

G6PDH is located in the pentose phosphate pathway in cells and serves to protect the cell from damage caused by oxidants. One hundred and fifty microliters of M1 was added to the cell culture medium, and the following methodology was used. The methodology that Olive et al. [42] recommended was used to determine the G6PDH level. According to this method, G6PDH activity in cell lysates was determined spectrophotometrically based on the rate of NADP^+^ reduction. Briefly, the reaction mixture contained Tris–HCl buffer (pH 7.8), glucose‐6‐phosphate as the substrate, NADP^+^ as the coenzyme, and an appropriate amount of sample. The reaction was initiated by the addition of glucose‐6‐phosphate, and the formation of NADPH was monitored by recording the increase in absorbance at 340 nm at 25°C. Enzyme activity was calculated using the molar extinction coefficient of NADPH and expressed as nanomoles of NADPH formed per minute per milligram of protein. Protein concentrations were determined prior to the assay, and all measurements were performed under conditions in which the reaction rate was linear with respect to time and protein concentration [42].

#### 2.7.4. Catalase (CAT) Determination

CAT is one of the enzymes that catalyze the splitting of hydrogen peroxide into water and molecular oxygen. One hundred microliters of M1 was taken up in cell culture, and the protocol was applied. CAT amount was determined according to the protocol recommended by Aebi [43]. According to the method described by Aebi, CAT activity in cell lysates was determined spectrophotometrically based on the rate of hydrogen peroxide (H_2_O_2_) decomposition catalyzed by CAT. The principle of the assay relies on the strong absorbance of H_2_O_2_ at 240 nm and the progressive decrease in absorbance as H_2_O_2_ is degraded by CAT. Briefly, the reaction mixture was prepared to contain phosphate buffer at an appropriate concentration (pH 7.0–7.4), a defined initial concentration of H_2_O_2_, and an appropriate amount of cell lysate. The reaction was initiated by the addition of the cell lysate, and the decrease in absorbance at 240 nm due to H_2_O_2_ degradation was monitored at 25°C over a defined period. CAT activity was calculated from the rate of change in absorbance (*Δ*A/min) using the molar extinction coefficient of H_2_O_2_ and was expressed as the amount of H_2_O_2_ decomposed per minute per milligram of protein. Protein concentrations were determined prior to the assay, and all measurements were performed under conditions in which the reaction rate was linear with respect to time and protein concentration [43].

#### 2.7.5. Lactate Dehydrogenase (LDH) Determination

It is the standard procedure for determining cytotoxicity in membrane integrity studies. The course of cytotoxicity caused by the molecule in cell cultures is quantitatively evaluated by measuring the activity of LDH in the supernatant. For 48 h, the cells were exposed to IC_50_ doses of M1 medication, and 100 *μ*L of M1 was taken in cell culture media. The methodology suggested by Decker and Lohmann‐Matthes [44] was used to assess LDH activity. LDH activity was experimentally determined as an indicator of cellular cytotoxicity based on the quantification of LDH released into the culture medium following cell damage. Briefly, after incubation of the cells under appropriate conditions, culture supernatants were collected and added to the reaction mixture for LDH analysis. The assay principle is based on the LDH‐catalyzed oxidation of lactate to pyruvate, accompanied by the reduction of NAD^+^ to NADH, which subsequently drives the reduction of a tetrazolium salt to form a colored formazan product. The intensity of the developed color was measured spectrophotometrically at a specific wavelength, and absorbance values were considered proportional to the amount of LDH released into the extracellular medium. LDH release was interpreted as a marker of compromised cell membrane integrity, and the results were expressed as percentage cytotoxicity or relative LDH activity compared with the control group. This experimental approach enables rapid and reliable quantitative assessment of cell death associated with cellular cytotoxicity [44].

#### 2.7.6. Superoxide dismutase (V) Determination

Sun et al. used a spectrophotometric apparatus to detect SOD activity, following the approach that was provided by [45]. SOD activity was determined using a spectrophotometric method based on the ability of SOD to inhibit reactions mediated by superoxide anions. The principle of the assay relies on the generation of superoxide radicals through the xanthine–xanthine oxidase system, which, under normal conditions, reduces an indicator compound, such as nitroblue tetrazolium (NBT). SOD present in the cell lysate catalyzes the dismutation of superoxide anions, thereby suppressing this reduction reaction. Briefly, cell lysates were added to the reaction mixture, and the color change resulting from NBT reduction was monitored spectrophotometrically at a specific wavelength. SOD activity was calculated based on the percentage inhibition of NBT reduction, and one unit of SOD activity was defined as the amount of enzyme required to produce 50% inhibition under the assay conditions [45].

### 2.8. Statistical Analysis

All data are expressed as mean ± SD. Statistical analyses were conducted using GraphPad Prism (Version 8, GraphPad Software, United States). For MTT time‐course experiments (24, 48, and 72 h), differences were evaluated using one‐way repeated‐measures ANOVA with Greenhouse–Geisser correction. Biochemical assay results and RT‐PCR data were compared between two groups using Student′s *t*‐test. For RT‐PCR, relative gene expression levels were calculated using the 2^−*Δ* *Δ*CT^ method, with CT values and fold‐change calculations performed using QIAGEN′s GeneGlobe RT^2^ Profiler PCR Data Analysis software. A *p* value < 0.05 was considered statistically significant.

### 2.9. Theoretical Methods

Theoretical simulations provide us with significant knowledge about the chemical and biological characteristics of molecules. Theoretical computations are used to derive a large number of quantum chemical parameters. The estimated values elucidate the chemical activities of the molecules. There are several different programs that may be used to compute molecules. The names of these applications are Gaussian09 RevD.01 and GaussView 6.0 [46, 47]. These programs were used to do computations utilizing the B3LYP, HF, and M062X [31–34] levels using the 6‐31++g(d,p) basis set. These computations have led to the discovery of a number of quantum chemical parameters. The computed parameters are determined in the following manner, and each parameter specifies a distinct chemical property of the molecules [48, 49].

χ=−∂Ε∂Νυr=12I+A≅12EHOMO+ELUMO,η=−∂2Ε∂Ν2υr=12I−A≅−12EHOMO−ELUMO,σ=1η ω=χ22η ε=1ω.

Molecular docking simulations are conducted to evaluate the chemical activities of the compounds in relation to the proteins. The Maestro (Version 12.8) [50] was utilized to perform the docking study. The process comprises several phases, with each being executed in a unique way. The first phase included the use of the protein preparation module [51], LigPrep [52], and Glide [53] to investigate the contacts that took place between the compounds and the target following the protein preparation. The OPLS4 force field was applied to perform the computations. Lastly, an ADME/T study will be performed to evaluate the pharmacological potential of the chemicals being investigated. The Qik‐prop module [54] of the Schrödinger program was used to forecast the effects and responses of compounds in human metabolism.

## 3. Results

### 3.1. In Vitro Assay for Cytotoxicity Activity (MTT Assay)

**Figure 1:**
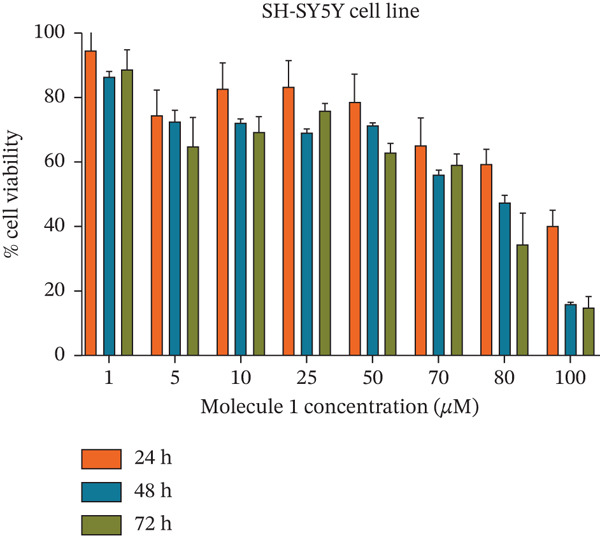
Cytotoxicity evaluation of M1 in SH‐SY5Y. The cell treatments were at 24, 48, and 72 h in a concentration range of 1–100 *μ*M. Shows the mean ± SEM derived from three distinct experiments.

SH‐SY5Y cells were treated with M1 at eight concentrations (1–100 *μ*M), and cell viability was assessed by the MTT assay after 24, 48, and 72 h. The IC_50_ values were 130.3 *μ*M (24 h), 51.87 *μ*M (48 h), and 30.50 *μ*M (72 h). Cytotoxic activity was determined by comparing M1‐treated cells with untreated control cells. As shown in Figure 1, M1 exhibited its strongest cytotoxic effect in SH‐SY5Y cells after 72 h of exposure (Table 1).

**Table 1:** Time‐dependent cytotoxic effect of M1 on SH‐SY5Y cells (MTT assay).

Parameter	24 h	48 h	72 h
Cell viability (%), mean ± SD	72.38 ± 17.07	61.43 ± 21.80	58.83 ± 23.60
95% CI of mean (%)	58.11–86.65	43.20–79.65	39.10–78.56
IC_50_ (*μ*M)	130.3	51.87	30.50
*F*(df)	*F*(1.866, 13.06) = 16.53		
*p* value	0.0003 ^∗^		
*R* ^2^	0.7025		

*Note:* One‐way repeated‐measures ANOVA with Greenhouse–Geisser correction.

^∗^ *p* < 0.05 (significant).

The cytotoxic effect of M1 on SH‐SY5Y cells was time‐dependent. MTT analysis showed a progressive reduction in cell viability from 24 to 72 h, with mean viabilities of 72.38*%* ± 17.07*%*, 61.43*%* ± 21.80*%*, and 58.83*%* ± 23.60*%* at 24, 48, and 72 h, respectively. Accordingly, IC_50_ values decreased from 130.3 *μ*M at 24 h to 51.87 *μ*M at 48 h and 30.50 *μ*M at 72 h. Repeated‐measures ANOVA with Greenhouse–Geisser correction confirmed a significant effect of incubation time on cell viability (*F*(1.866, 13.06) = 16.53, *p* = 0.0003, *R* ^2^ = 0.7025), indicating a statistically significant time‐dependent cytotoxic response to M1 (Table 1).

### 3.2. Cell Morphology Analysis

**Figure 2:**
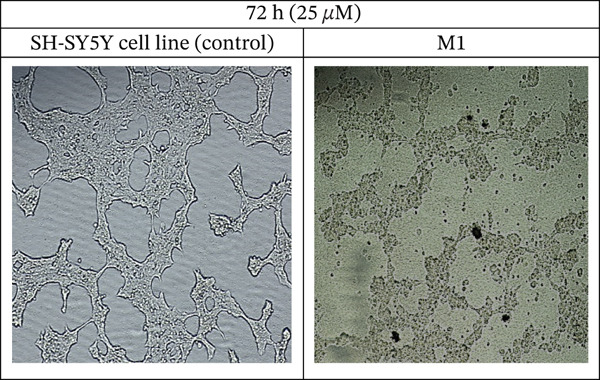
Morphological alterations of SH‐SY5Y cells following 72 h of incubation with a concentration of 25 *μ*M of M1 are reported, based on microscopic observations and photography. Control and M1‐treated cells were examined and photographed under a microscope (Olympus BX 51) using a 40× objective.

Figure 2 shows that morphological analyses in SH‐SY5Y were conducted following the administration of 25 *μ*M M1 for 72 h. It was shown that the morphology of M1 was notably distinct from that of the SH‐SY5Y control group.

### 3.3. Gene Expression Analysis

**Figure 3:**
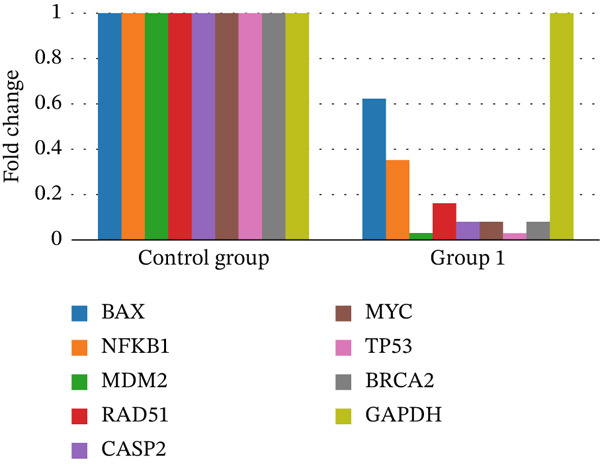
The gene expression of *MYC*, *CASP2*, *TP53*, *RAD51*, *BRCA2*, *MDM2*, *BAX*, and *NF-κB1* in the groups.

In the present study, the expression profiles of apoptosis‐related genes (*MYC*, *CASP2*, *BAX*, and *NF-κB1*) and cell cycle/DNA repair–related genes (*TP53*, *RAD51*, *BRCA2*, and *MDM2*) were quantified in SH‐SY5Y cells following treatment with the test molecule (Group 1, M1) using RT‐PCR. CT values and relative expression levels were processed using RT^2^ Profiler RT‐PCR Sequence Data Analysis v3.5 and Rotor‐Gene 6000 software [48]. As shown in Figure 3, all target genes differed significantly between Group 1 and the control group (Student′s *t*‐test, *n* = 3; *p* < 0.0001). Compared with control, *BAX* and *NF-κB1* exhibited modest downregulation (fold regulation: −1.61 and −2.85, respectively), whereas stronger regulation was observed for *MDM2* (−34.78) and *TP53* (−30.06), along with substantial changes in *CASP2* (−12.73), *MYC* (−12.04), *BRCA2* (−12.82), and *RAD51* (−6.19). *GAPDH* remained stable (fold regulation = 1.00), confirming its suitability as the reference gene (Table 2).

**Table 2:** qPCR results showing CT values, fold regulation, and statistical differences among experimental groups in the SH‐SY5Y cell lines.

Genes	Groups	Mean *C* *T*	Fold regulation	*p* value
*BAX*	Group 1	23.40	−1.61	< 0.0001 ^∗^
	Control	25.43		
*NF-κB1*	Group 1	23.02	−2.85	< 0.0001 ^∗^
	Control	24.23		
*MDM2*	Group 1	24.27	−34.78	< 0.0001 ^∗^
	Control	21.87		
*RAD51*	Group 1	22.77	−6.19	< 0.0001 ^∗^
	Control	22.86		
*CASP2*	Group 1	24.98	−12.73	< 0.0001 ^∗^
	Control	24.03		
*MYC*	Group 1	27.48	−12.04	< 0.0001 ^∗^
	Control	26.61		
*TP53*	Group 1	26.23	−30.06	< 0.0001 ^∗^
	Control	24.04		
*BRCA2*	Group 1	25.52	−12.82	< 0.0001 ^∗^
	Control	24.56		
*GAPDH*	Group 1	21.31	1.00	N/A
	Control	24.03		

*Note:* Statistical comparisons were performed using Student′s *t*‐test.

Abbreviations: CT, cycle threshold; GAPDH, glyceraldehyde 3‐phosphate dehydrogenase.

^∗^ *p* < 0.0001 (significant).

### 3.4. Biochemical Analyses

To further characterize the cellular response to M1, intracellular antioxidant status and enzyme activities (GSH, GST, G6PDH, CAT, SOD, and LDH) were evaluated in SH‐SY5Y cells following exposure to M1 at its IC_50_ concentration for 48 h. All biochemical assays were performed in triplicate (*n* = 3). Biochemical parameters (GSH, SOD, GST, G6PDH, CAT, and LDH) were compared between the control and M1‐treated groups using Student′s *t*‐test. GSH, SOD, and GST activities were significantly increased in the M1‐treated group (*p* < 0.05), whereas no significant differences were observed for G6PDH, CAT, or LDH (*p* > 0.05) (Table 3).

**Table 3:** Effect of M1 on antioxidant enzymes and LDH activity in SH‐SY5Y cells.

Parameter	Control (%)	M1 (%)	95% CI of M1 (%)	*p* value
GSH	100	103.4	59.93–146.8	0.0210 ^∗^
SOD	100	108.5	0.497–216.5	0.0498 ^∗^
GST	100	97.0	58.88–135.1	0.0197 ^∗^
G6PDH	100	116.5	−80.45 to 313.4	0.0842
CAT	100	139.0	−356.5 to 634.5	0.1741
LDH	100	111.5	−34.62 to 257.6	0.0654

*Note:* Data were normalized to the control (100%). Values are presented as means with 95% confidence intervals. Statistical comparisons were performed using Student′s *t*‐test.

^∗^ *p* < 0.05 (significant).

#### 3.4.1. Glutathione Determination

**Figure 4:**
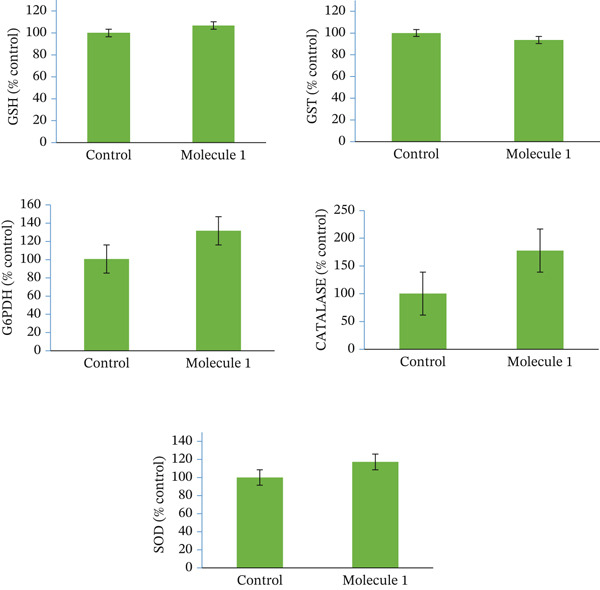
GSH, GST, G6PDH, CAT, and SOD levels of M1 in SH‐SY5Y cell lines. The assessment of GSH, GST, G6PDH, SOD, and CAT levels also indicates the antioxidant capacity.

SH‐SY5Y cell lines treated with the M1 molecule were incubated with IC_50_ concentrations for 48 h. A slight increase in GSH levels was observed in the SH‐SY5Y when this molecule was compared to the control in Figure 4.

#### 3.4.2. Glutathione‐S‐Transferase Determination

The GST activity of M1 in the SH‐SY5Y cell line was decreased in comparison to the control in Figure 4.

#### 3.4.3. Glucose‐6‐Phosphate Dehydrogenase Determination

It was determined that M1 had a higher effect on the G6PDH enzyme activity in the SH‐SY5Y cells in comparison to the control in Figure 4.

#### 3.4.4. Catalase Determination

In Figure 4, the SH‐SY5Y cell line′s CAT activity rose by 78% in comparison to the control.

#### 3.4.5. Determination of Superoxide Dismutase

In Figure 4, the SOD enzyme activity of the molecule was somewhat higher in the SH‐SY5Y than in the control.

#### 3.4.6. Superoxide Dismutase Determination

**Figure 5:**
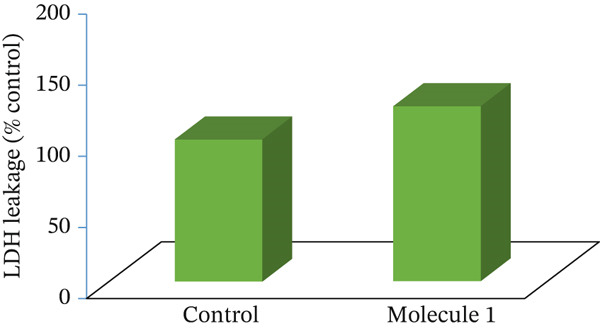
LDH releases of M1 from SH‐SY5Y after 48‐h incubation at IC_50_ concentration. Another measure of membrane integrity is LDH activity.

Comparing the SH‐SY5Y cell line to the control group in Figure 5, it was found that the M1 medication dramatically decreased the cell survival rate.

The study′s findings showed that the cytotoxic activity of the M1 medication was at its highest in SH‐SY5Y cells after 72 h of incubation. In parallel, the experimental data indicated that M1 modulated cellular programs linked to antioxidant defense, oxidative stress control, detoxification, and survival. Since alterations in apoptosis and cell cycle–related gene expression can markedly affect the viability and behavior of SH‐SY5Y NB cells [55, 56], we further investigated how M1 influences these transcriptional responses.

A major contributor to chemo‐ and radioresistance in many tumors is disruption of the p53 tumor suppressor pathway [57]. p53 regulates stress responses and can activate apoptosis‐related genes such as MDM2 and BAX, with MDM2 forming a negative feedback loop that tightly controls p53 activity [57]. In some cellular contexts, p53 promotes BAX transcription, facilitating mitochondrial permeabilization, cytochrome c release, caspase activation, and apoptotic cell death [58]. Notably, TP53 mutations are uncommon at diagnosis but have been reported in NB cell lines derived at relapse or after therapy, where they may be associated with acquired drug resistance [59, 60].

In our investigation, M1 treatment was associated with lower *TP53* expression in SH‐SY5Y cells compared to the control group, accompanied by reduced *MDM2* and *BAX* expression. Despite this overall decrease, *BAX* expression remained relatively higher than that of the other analyzed genes, which may indicate persistence of proapoptotic signaling within the tested gene panel under M1 exposure.

*MYC* is overexpressed in approximately 20% of NB cases and is associated with a high‐risk phenotype, in part through broad changes in gene regulation [61]. Elevated *MYC* expression may promote reactive oxygen species (ROS) accumulation, thereby increasing DNA damage and mutational pressure [62], and has been linked to mitochondrial and antioxidant‐related processes [63, 64]. MYC also supports survival under repeated DNA damage by engaging repair programs and interacting with promoter regions of double‐strand break repair genes such as NBS1, KU70, RAD51, BRCA2, and RAD50 [65]. In our study, M1 decreased MYC expression together with the DNA repair–associated genes RAD51 and BRCA2 in SH‐SY5Y cells, suggesting that M1 may attenuate MYC‐linked adaptive repair capacity, which could contribute to the higher cytotoxicity observed at 72 h.

In NB biology, MYC overexpression is widely linked to a more aggressive, high‐risk phenotype and enhanced stress tolerance [66]. From this perspective, the concurrent reduction of MYC together with key DNA repair genes such as RAD51 and BRCA2 may suggest that M1 weakens a core survival program that helps SH‐SY5Y cells cope with repeated DNA damage [63]. In practical terms, limiting these repair and adaptation routes could make the cells less able to recover from oxidative or genotoxic stress, which aligns well with the stronger cytotoxic effect we observed at 72 h. At the same time, the altered TP53–MDM2–BAX pattern may reflect a reshaping of stress‐response and apoptosis signaling pathways that are often disrupted or rewired during NB progression and treatment resistance [55, 56, 59, 67].

Metabolic adaptation is a key feature enabling malignant cells to withstand stressors such as ROS overload and DNA damage. GSH plays a central role in redox buffering, detoxification, and resistance mechanisms, and elevated GSH levels in cancer cells have been associated with increased tolerance to chemotherapy in multiple tumor types [68, 69]. In our investigation, a slight increase in GSH levels was detected in SH‐SY5Y cells following M1 treatment, which may reflect an adaptive antioxidant response to treatment‐induced stress. Consistently, CAT, whose expression and activity can be strongly influenced by cellular metabolic context and tumor microenvironmental cues, has been reported to be overexpressed in certain cancers and associated with prognosis [70, 71]. In the present study, M1 increased CAT activity in SH‐SY5Y cells compared to controls. Alongside these antioxidant‐related changes, LDH, a commonly used indicator of membrane integrity and cytotoxicity in cell culture, can rise in response to treatment‐related cellular injury [72, 73]. Here, LDH activity increased in M1‐treated SH‐SY5Y cells compared to controls, suggesting a potential contribution of membrane damage to the observed cytotoxicity. Finally, SOD is a primary enzymatic defense against superoxide radicals generated during mitochondrial respiration, and sustained ROS accumulation can trigger oxidative stress and damage critical biomolecules [74]. In our research, SOD activity also increased in SH‐SY5Y cells under M1 exposure, further supporting the involvement of redox stress responses.

Collectively, the experimental profile peak in cytotoxicity at 72 h, accompanied by coordinated changes in apoptosis/cell cycle–related genes, MYC‐linked repair transcripts (RAD51/BRCA2), and redox/oxidative stress markers (GSH, CAT, LDH, SOD), is consistent with a scenario in which M1 perturbs cellular stress adaptation and survival programs. These observations can be interpreted together with the theoretical calculations (Table 2) and docking analyses (Table 3), where the quantum chemical descriptors and protein interaction patterns provide a mechanistic framework to rationalize how M1′s physicochemical reactivity and target affinity may translate into the experimentally observed effects on oxidative stress handling and survival‐associated pathways. In particular, the stronger docking preference observed for the targets represented by 5WIV and 3PBL (Table 3) may help explain the experimental pattern of altered stress‐response signaling and reduced MYC‐linked repair capacity, although target engagement studies are needed for confirmation.

### 3.5. Theoretical Methods

Comparing the activity of molecules using theoretical calculations is quick and easy. The Gaussian package software has been used in a number of quantum chemistry computations that include molecules. The lowest unoccupied molecular orbital (LUMO) and the highest occupied molecular orbital (HOMO) are the most important of these approximated quantum chemical characteristics. These parameters have been utilized to interpret the compounds′ activities and describe their electron‐donating properties. The molecule with the greater activity level is the one with the greatest positive value for the HOMO parameter [75]. However, the most active molecule has the lowest energy empty molecular orbital characteristic, which has the biggest negative numerical value when compared to other molecules [76]. A number of parameters were discovered from the calculations. Refer to Table 4 for each of these parameters.

**Table 4:** The calculated quantum chemical parameters of the molecule.

	*E*_*H* *O* *M* *O*_	*E*_*L* *U* *M* *O*_	*I*	*A*	*Δ* *E*	*η*	*Μ*	*X*	PA	*ω*	*ε*	Dipole	Energy
B3LYP/6‐31g level	−6.2712	−2.0134	6.2712	2.0134	4.2578	2.1289	0.4697	4.1423	−4.1423	4.0299	0.2481	3.2700	−40,331.3605
B3LYP/6‐31++g level	−6.5787	−2.3641	6.5787	2.3641	4.2145	2.1073	0.4745	4.4714	−4.4714	4.7439	0.2108	3.5663	−40,333.0552
B3LYP/6‐31++g(d,p) level	−6.5648	−2.3018	6.5648	2.3018	4.2630	2.1315	0.4692	4.4333	−4.4333	4.6105	0.2169	3.1710	−40,345.3553
HF/6‐31 g level	−8.8748	1.8104	8.8748	−1.8104	10.6852	5.3426	0.1872	3.5322	−3.5322	1.1676	0.8564	3.2550	−40,076.1648
HF/6‐31++g level	−9.0166	0.8710	9.0166	−0.8710	9.8876	4.9438	0.2023	4.0728	−4.0728	1.6776	0.5961	3.2908	−40,077.3666
HF/6‐31++g(d,p) level	−9.0038	0.8964	9.0038	−0.8964	9.9001	4.9501	0.2020	4.0537	−4.0537	1.6598	0.6025	2.6545	−40,095.4376
M062X/6‐31g level	−7.5866	−1.0841	7.5866	1.0841	6.5025	3.2512	0.3076	4.3354	−4.3354	2.8905	0.3460	4.0096	−40,316.0851
M062X/6‐31++g level	−7.8533	−1.3968	7.8533	1.3968	6.4565	3.2282	0.3098	4.6250	−4.6250	3.3131	0.3018	3.4610	−40,317.5732
M062X/6‐31++g(d,p) level	−7.8342	−1.3279	7.8342	1.3279	6.5063	3.2531	0.3074	4.5811	−4.5811	3.2255	0.3100	3.0611	‐40,329.2752

**Figure 6:**
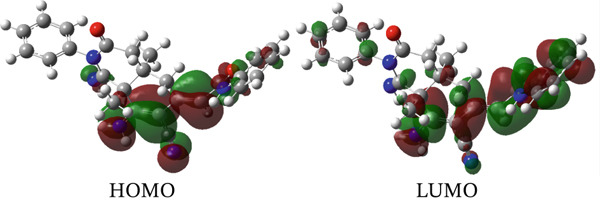
Representations of HOMO and LUMO of M1.

**Figure 7:**
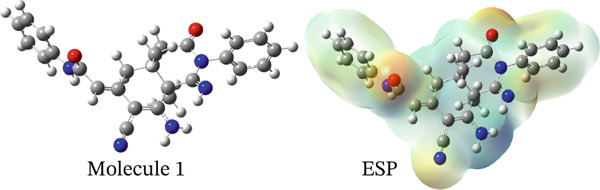
Representations of optimize structure and ESP of M1.

When contrasting molecules′ activities, a variety of quantum chemical parameters are taken into consideration, including the following: chemical hardness, softness, electronegativity, chemical potential, *Δ* *E*, *E*_HOMO_, and *E*_LUMO_. These characteristics are linked to a compound′s ability to provide or receive electrons. In addition, the numerical values of these parameters may be calculated using Koopman′s theorem [77]. It provides an alternative method for utilizing boundary orbital energies to ascertain the ionization energy and electron affinity of a compound (Figures 6 and 7).

Chemical hardness describes the resistance of a compound to deformation or polarization of its electron cloud and was introduced by Pearson [78] within the framework of Lewis acid–base theory. This concept forms the basis of the hard and soft acid–base (HSAB) principle [79], which classifies acids and bases as hard or soft and states that hard acids preferentially interact with hard bases, whereas soft acids favor soft bases. The maximum hardness principle (PMH) [80] further suggests that chemical systems tend to adopt states of maximum hardness. In this context, hardness is associated with low polarizability, while softness corresponds to easily polarizable species with enhanced electron‐donating ability. According to Koopmans′ theorem [77], chemical hardness is directly related to the HOMO–LUMO energy gap, where a larger gap indicates a harder and more stable molecule, and a smaller gap reflects a softer and more reactive structure. Since softness (1/*η*) represents the inverse of hardness, soft molecules exhibit higher polarizability and greater reactivity due to their increased capacity for electron transfer [81].

**Figure 8:**
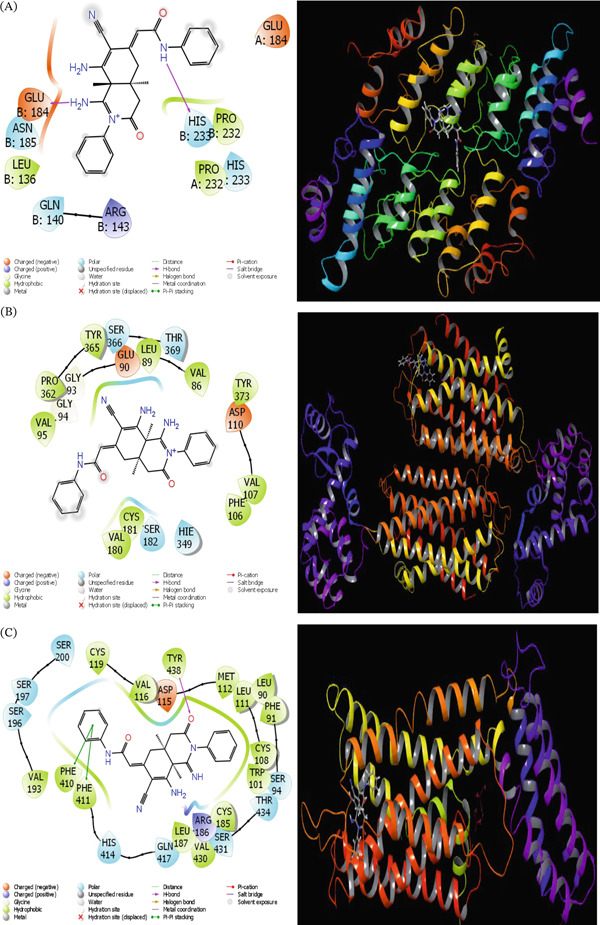
(A) The interactions and pose of M1 with the 2F37 protein. (B) The interactions and pose of M1 with the 3PBL protein. (C) The interactions and pose of M1 with the 5WIV protein.

Theoretical simulations are an important way to compare molecular activity; however, the Gaussian package software by itself is not enough to do this job. This is due to the fact that Gaussian calculations only consider molecules, but other theoretical calculations do not account for how molecules react to other biological components. Molecular docking calculations are the most often utilized method for these simulations [82]. The numerical value of the docking score, which is an essential parameter, is determined by the affinity of molecules to proteins in Figure 8 [83, 84]. These include hydrogen bonds, *π*–*π*, halogen, polar, and hydrophobic interactions, as well as other types of chemical interactions [85, 86]. The molecules become more active due to the improved interactions between the proteins and the molecules. Figure 8A–C provides a thorough analysis of the interactions that take place between proteins and substances. Table 5 displays all of the parameters that were acquired.

**Table 5:** Parameters obtained from docking calculation.

	2F37		3PBL		5WIV	
	*M*1	*C*	*M*1	*C*	*M*1	*C*
Docking score	−2.99	−6.25	−5.24	−5.24	−8.28	−7.86
Glide ligand efficiency	−0.09	−0.26	−0.16	−0.22	−0.25	−0.33
Glide hbond	−0.22	−0.75	−0.25	−0.56	−0.32	−0.62
Glide evdw	−27.35	−26.52	−34.01	−28.31	−50.88	−38.36
Glide ecoul	−9.43	−14.77	−10.89	−11.73	−3.85	−6.12
Glide emodel	−43.65	−46.27	−62.58	−59.88	−49.03	−60.41
Glide energy	−36.78	−41.29	−44.90	−40.04	−54.73	−44.48
Glide einternal	6.40	17.04	1.18	7.91	13.56	8.75
Glide posenum	287	29	17	377	9	104

Abbreviation: *C*, ampicillin.

The two most significant elements that were found in molecular docking examinations of the glide emodel and docking score parameters are molecules that target different proteins. Important details on the biological activities of molecules can be found in the numerical values of these attributes. The chemical with the lowest values for the glide emodel parameters and docking score is the one that exhibits the most biological activity against that protein. The high level of biological activity of substances against proteins suggests that there is a significant amount of interaction between the two. It is well recognized that as the number of molecular interactions with proteins increases, so does the number of attachments. As a consequence, the biological activity of the molecule rises.

Ligand Glide Another statistic that comes from molecular docking calculations is efficiency, which expresses how effective the chemical is against proteins [48]. Another parameter called glide hbond provides a numerical representation of the number of hydrogen bonds formed during interactions between the chemical and proteins. The van der Waals interaction number, also referred to as glide evdw, is another metric that shows how chemicals and proteins interact. An extra parameter is glide ecoul, which is the numerical description of the Coulomb interactions that occur between compounds and proteins. Glide einternal, a number that is the outcome of mixing multiple parameters, is the final parameter derived from these computations [87].

According to the MM‐GBSA results given in Table 6, the binding of the M1 molecule to the 5WIV protein is thermodynamically favorable, and the total binding free energy (*Δ* *G* bind) was calculated as −38.16 kcal/mol. This value indicates that complex formation is stable and spontaneous. When the energy breakdown is examined, it is seen that the binding is primarily driven by van der Waals (−47.86 kcal/mol) and lipophilic interactions (−33.25 kcal/mol); this indicates that the ligand develops a good shape fit to the active site and strong hydrophobic contacts.

**Table 6:** MM‐GBSA parameters of the M1 molecule with the 5WIV protein.

MM‐GBSA dG bind	−38.16
MM‐GBSA dG bind Coulomb	−5.96
MM‐GBSA dG bind covalent	15.58
MM‐GBSA dG bind Hbond	−0.42
MM‐GBSA dG bind Lipo	−33.25
MM‐GBSA dG bind packing	−2.89
MM‐GBSA dG bind Solv GB	36.64
MM‐GBSA dG bind vdW	−47.86

Electrostatic contribution (Coulomb, −5.96 kcal/mol) supports binding, while polar solvent effect (Solv GB, +36.64 kcal/mol) constitutes a significant desolvation penalty. The contribution of hydrogen bonds is quite limited (−0.42 kcal/mol), suggesting that binding is mainly based on nonpolar interactions. The positive term “covalent” (+15.58 kcal/mol) refers to the conformational or intrinsic energy cost during complex formation. Overall, the results reveal that M1 is stabilized in the 5WIV binding pocket by strong hydrophobic and dispersion‐based interactions.

The observed cytotoxic activity can be rationalized based on the structural features of the synthesized compounds. The presence of electron‐donating and electron‐withdrawing substituents on the aromatic ring significantly influences the electronic distribution and consequently modulates the biological activity. Compounds bearing electron‐withdrawing groups exhibited enhanced cytotoxicity, which may be attributed to increased electrophilic character and improved interaction with nucleophilic sites in biomacromolecules. Additionally, the conjugated *π* system within the molecular backbone enhances planarity and facilitates *π*–*π* stacking interactions with aromatic amino acid residues in target proteins, thereby strengthening binding affinity.

Furthermore, the incorporation of the metal center (or nanoparticle/ferrocene moiety, if applicable) contributes to redox activity and may promote ROS generation, which is known to induce apoptosis in cancer cells. Increased lipophilicity of certain derivatives may also improve membrane permeability, leading to higher intracellular accumulation and enhanced cytotoxic response. The lower IC_50_ values observed for specific derivatives suggest that structural modifications affecting electronic properties, steric hindrance, and hydrophobic balance play a crucial role in determining anticancer efficacy. Overall, the cytotoxicity trend correlates well with the electronic and steric characteristics of the synthesized molecules, highlighting a clear structure–activity relationship.

A chemical with high activity does not necessarily create a good molecule if it is only studied in relation to molecules and proteins. This calls for a comprehensive analysis of the chemicals utilizing ADME/T. The results of the ADME/T calculations for substances, which examine both their chemical and biological properties, are displayed in Table 7.

**Table 7:** ADME properties of the molecule.

	Molecule	Reference range
mol_MW	440	130–725
Dipole (*D*)	7.2	1.0–12.5
SASA	690	300–1000
FOSA	98	0–750
FISA	200	7–330
PISA	392	0–450
WPSA	0	0–175
Volume (*A* ^3^)	1313	500–2000
donorHB	3.5	0–6
acceptHB	9	2.0–20.0
glob (sphere = 1)	0.8	0.75–0.95
QPpolrz (*A* ^3^)	46.3	13.0–70.0
QPlogPC16	15.4	4.0–18.0
QplogPoct	26.5	8.0–35.0
QplogPw	18.1	4.0–45.0
QPlogPo/w	2.6	−2.0 to 6.5
QplogS	−5.6	−6.5 to 0.5
CIQPlogS	−6.5	−6.5 to 0.5
QPlogHERG	−6.0	^a^
QPPCaco (nm/s)	125	^b^
QplogBB	−1.7	−3.0 to 1.2
QPPMDCK (nm/s)	52	^b^
QplogKp	−3.3	Kp in cm/h
IP (eV)	8.9	7.9–10.5
EA (eV)	0.9	−0.9 to 1.7
#metab	4	1–8
QplogKhsa	0.2	−1.5 to 1.5
Human oral absorption	3	—
Percent human oral absorption	80	^c^
PSA	126	7–200
RuleOfFive	0	Maximum is 4
RuleOfThree	0	Maximum is 3
Jm	0.0	—

^a^Corcern below −5.

^b^< 25 is poor, and > 500 is great.

^c^< 25% is poor, and > 80% is high.

Various characteristics, including molecular mass, dipole moment, volume, total solvent accessible surface area (SASA), and the number of hydrogen bonds given or received, are critical characteristics of molecules. Nevertheless, considering the biological characteristics of the molecules, the anticipated IC_50_ for HERG K+ channel inhibition, which serves as a model for the gut‐blood barrier, the prediction of the brain/blood partition coefficient, the estimated skin permeability, the unanticipated apparent MDCK cell diffusion, the forecast of binding to human serum albumin, and the predicted qualitative characteristics of human oral absorption are among the various parameters that have been computed [88].

Their amounts should be in the criteria in order for these traits to be assigned to different organs. Additionally, two characteristics, RuleOfFive [89, 90] (number of violations of Lipinski′s rule of five) and RuleOfThree [91] (number of violations of Jorgensen′s rule of three), are evaluated quantitatively to ascertain whether a compound satisfies the criteria for being categorized as a drug. It is predicted that the number of these two characters will be zero.

## 4. Conclusion

Collectively, the present data demonstrate that M1 exerts anticancer effects in SH‐SY5Y NB cells and is associated with significant modulation of apoptosis and cell cycle/DNA repair–related gene expression, together with alterations in enzyme activity and redox/oxidative stress markers. Complementary in silico analyses supported these observations: Quantum chemical calculations (B3LYP, HF, and M062X) coupled with ESP mapping delineated plausible reactive regions, and molecular docking predicted the strongest interaction with 5WIV, followed by 3PBL and 2F37. Moreover, ADME/T profiling indicated acceptable drug‐like characteristics within the applied criteria. These findings provide a rationale for subsequent studies to verify M1′s mechanism of action and molecular targets and to evaluate its efficacy and safety in advanced in vitro platforms and in vivo models.

## Author Contributions

**Tugba Agbektas:** investigation, formal analysis, conceptualization, writing – review and editing. **Ayca Tas:** investigation, formal analysis, conceptualization, writing – review and editing. **Cemile Zontul:** investigation, formal analysis, conceptualization, writing – review and editing. **Alireza Poustforoosh:** investigation, formal analysis, conceptualization, writing – review and editing. **Farid N. Naghiyev:** methodology, writing – original draft, writing – review and editing. **Ali N. Khalilov:** methodology, writing – original draft, writing – review and editing. **Unal Ozum:** methodology, writing – original draft, writing – review and editing. **Burak Tüzün:** supervision, formal analysis, conceptualization, methodology, writing – original draft, writing – review and editing. **Yavuz Silig:** supervision, formal analysis, conceptualization, methodology, writing – original draft, writing – review and editing. **Ibrahim G. Mamedov:** supervision, formal analysis, conceptualization, methodology, writing – original draft, writing – review and editing.

## Funding

This study was funded by the Scientific Research Project Fund of Sivas Cumhuriyet University (CUBAP), RGD‐020, and the Baku State University, BSU50/50.

## Conflicts of Interest

The authors declare no conflicts of interest.

## Supporting information


**Supporting Information** Additional supporting information can be found online in the Supporting Information section. Data associated with this article include additional figures, tables, and computational details that support the findings presented in the main text. These materials are available in the supporting information file.

## Data Availability

The authors confirm that the data supporting the findings of this study are available within the article and/or its supporting information.
