# Supplementary material for: Comprehensive Experimental and Computational Characterization of a Phenylacetamide‐Based Molecule
Source: Biomed Res Int. 2026 Jul 14;2026:2415605. doi: 10.1155/bmri/2415605 (PMC13366780; doi:10.1155/bmri/2415605)
Supplement: Supplementary file 1 — Supporting Information Additional supporting information can be found online in the Supporting Information section. Data associated with this article include additional figures, tables, and computational details that support the findings presented in the main text. These materials are available in the supporting information file. [file BMRI-2026-2415605-s001.docx]

**Supplementary information**


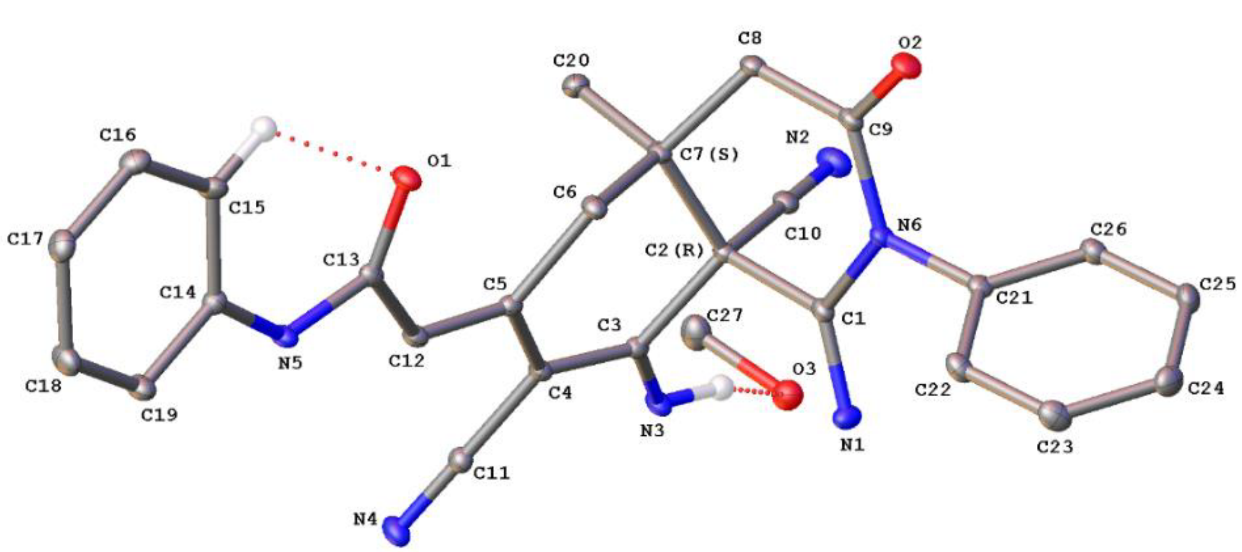


**Figure S1.** ORTEP plots for molecule **1** [57]


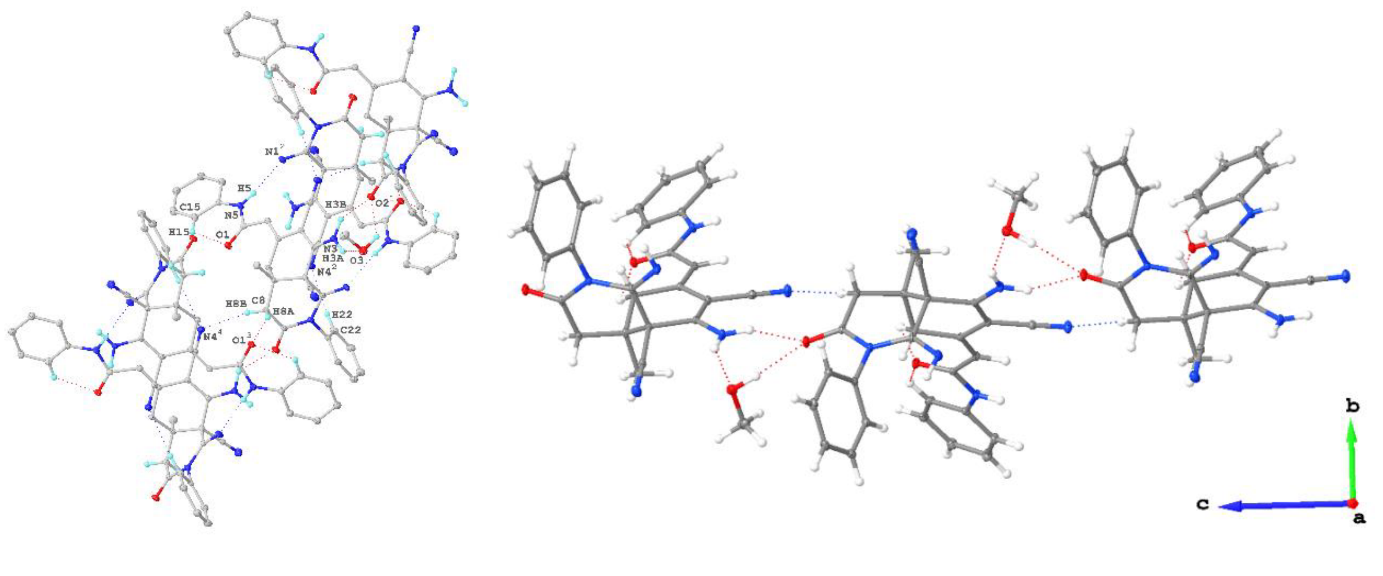


**Figure S2.** Intermolecular interactions observed in molecule **1** [57]


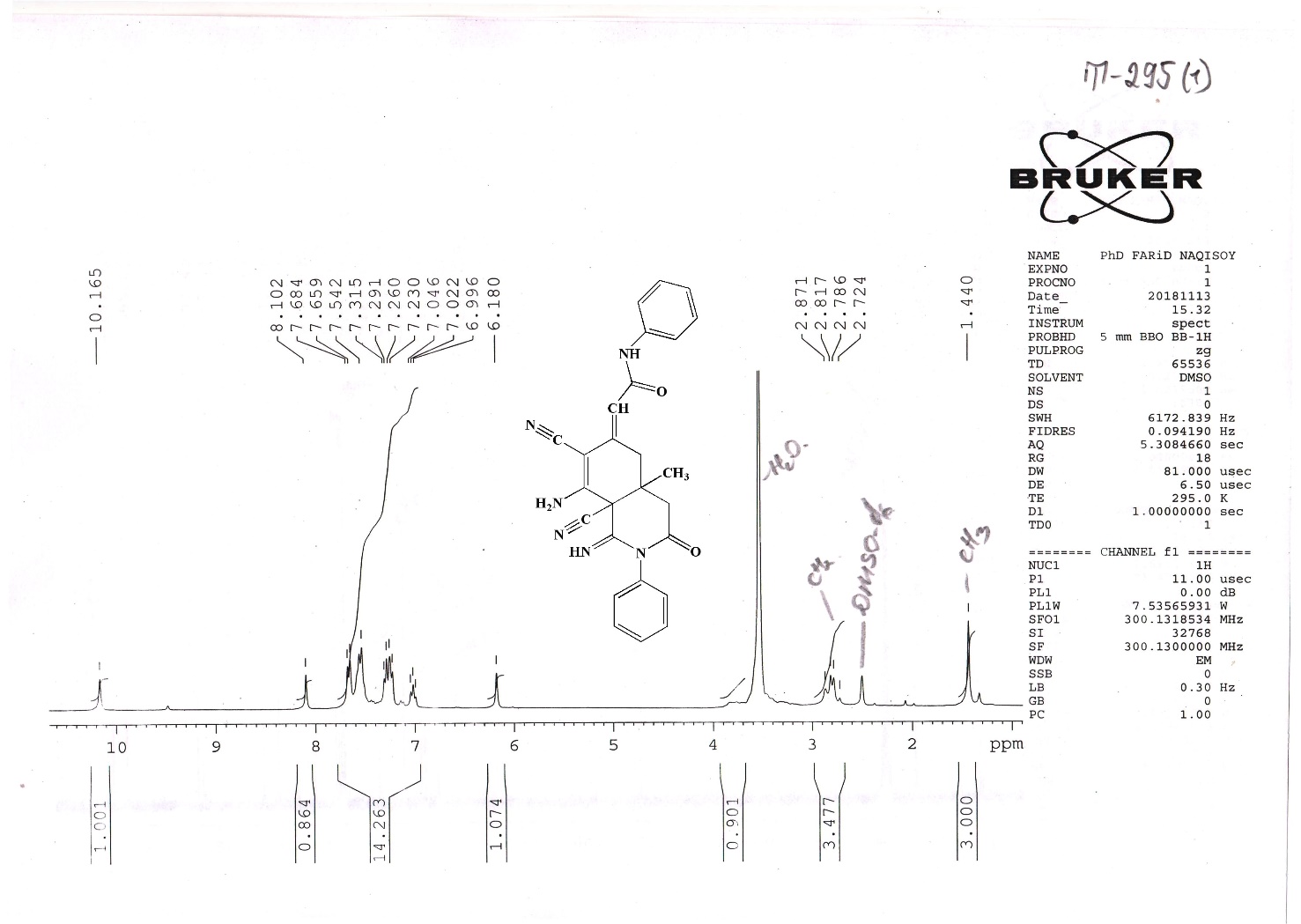


**Figure S3.** The ^1^H NMR spectrum of molecule **1** [57].


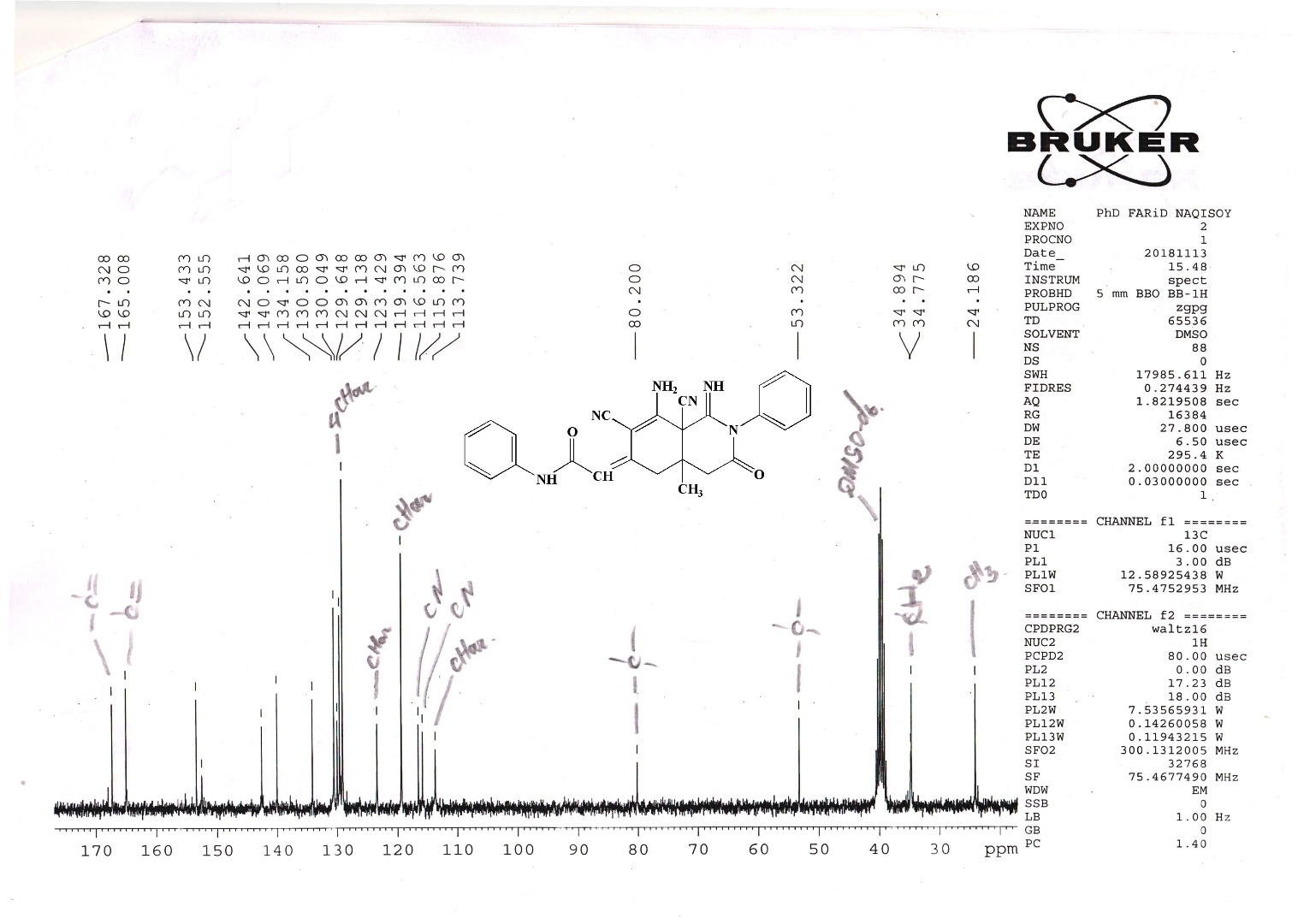


**Figure S4.** The ^1^H NMR spectrum of molecule **1**.
